# Ultrasound-guided Central Venous Catheterization for Home Parenteral Nutrition and Hydratation in Advanced Incurable Cancer Patients: Results of A Prospective Observational Study

**DOI:** 10.4021/wjon390e

**Published:** 2011-10-28

**Authors:** Luigi Cavanna, Maria Rosa Cordani, Claudia Biasini, Camilla Di Nunzio, Michela Monfredo, Elisa Stroppa, Monica Muroni, Massimo Ambroggi, Lara Muroni, Roberto Di Cicilia, Gabriele Cremona, Elisabetta Nobili, Elena Zaffignani, Giuseppe Civardi

**Affiliations:** a Azienda Ospedaliera “Guglielmo da Saliceto”, Oncology and Hematology Department, Oncology Unit, Via Taverna 49, 29100 Piacenza, Italy

**Keywords:** Central venous catheter, Palliative care, Cancer, Ultrasound, Nutrition, Hydratation

## Abstract

**Background:**

Most patients with advanced cancer are frequently malnourished and frequently they develop decreased oral fluid intake and dehidratation. Home parenteral nutrition (HPN) is an increasingly used therapy for patients with advanced cancer. A central venous access device is often an essential component allowing parenteral nutrition and hidratation. However central venous catheter (CVC) insertion represents a risk for pneumothorax or other mechanical complications. This study aimed to determine the reduction of risks related to central venous catheter positionement in the setting of cancer patients with palliative programm.

**Methods:**

Consecutive patients with a variety of cancer in advanced phase requiring palliative care who were undergoing placement of central venous catheter for parenteral nutrition or hydratation have been prospectively studied in a program of ultrasound-guided CVC placement. Four types of possible complications were defined:mechanical, thrombotic, infection and malfunctioning. After sterilization, local anesthesia is applied and a 7.5 MHZ puncturing US probe is placed in the supraclavicular site and a 16-gauge needle is advanced under real-time US guidance, into the last portion of internal jugular vein by experienced physicians. The Seldinger tecnique is used to place the catheter that is advanced into the superior vena cava until insertion to right atrium. Two hours after each procedure a chest X-ray and US scanning are carried out to confirm CVC position and rule out a pneumotorax.

**Results:**

From 30 October 2000 to 31 October 2008: 209 CVC insertional procedure were applied in 207 patients with cancer in the palliative phase only. There were 101 women and 106 men with a mean age of 67.68 year (range 22-86). A single needle puncture of the vein was performed on 206 of 209 procedures (98.6%), the technique was efficacious at the first attempt in 98.6% of cases, in 2 patients (0.96%) the CVC was positioned at the second attempt. The procedure failed only one case (0.44%). No cases of pneumothorax, of major bleeding or nerve punctured were reported. Symptomatic vein thrombosis developed in one patient (0.44%). Infection episodes were reported in two cases. Mean time for CVC permanence was 92.5±9.1 days (range 8-158).

**Conclusion:**

This study indicates that US-guided CVC insertion is a safe, cheap procedure for cancer patients in advanced phase and with palliative program, allowing parenteral nutrition and hydratation.

## Introduction

Patients with advanced cancer are frequently malnourished and dehidratated and, in some cases, these are the dominant symptoms for their disease [1]. For such patients, nutritional support seems not only logical, but also humane [1], and adequate vascular access is frequently essential for parenteral nutrition, administration of fluids or medication. The percutaneous approach to the subclavian or internal jugular vein currently is the most popular procedure for placing catheters in the superior vena cava both for short-term and long-term use.

Unfortunately, central venous catheter (CVC) insertion represents a risk for pneumothorax, nerve puncture and major bleeding (mechanical complications), infection and CVC-related vein thrombosis [2, 3].

Mechanical complications, of CVC insertion without ultrasound (US) guidance, such as arterial puncture and pneumothorax are seen up to 21% and up to 35% of insertion attempts are not successful [4-6].

There have been several prospective randomized trials [7-15] and two metanalyses [16, 17] that suggest the use of US has been associated with a reduction in complication rate and an improved firs-pass success when placing CVC in the internal jugular vein.

In 2001, the Agency for Healthcare Research and Quality from USA recommended the use of US for the placement of CVC, as one of their 11 practices to improve patient care [18, 19]. However a survey of 250 anesthetists in the United Kindom found that 41% disagreeded or strongly disagreeded with the recommendation that ultrasound imaging should be the preferred method for insertion of a central venous catheter in the internal jugular vein [20].

In addition a report in the United States also showed that < 15% of surgery, anesthesia, internal medicin, emergency medicine and family medicine housestaff used ultrasound guidance for most CVC placements [21].

Since in our department, oncologists and hematologists perform ultrasound imaging procedures as well as interventional ultrasound in clinical practice for patients management (diagnosis, staging, restaging and follow-up), from almost 25 years [22-28], this procedure was early applied as a guidance for CVC insertion in the internal jugular vein in cancer patients before availability of the recommendations from the Agency for Healthcare Research and Quality [18, 19], and recently we reported the results of a prospective observational study of 1978 consecutive central venous catheterisations in cancer patients undergoing chemotherapy or bone marrow transplantation [28]. However reports of central venous catheters-related complications in patients with palliative care program only are fragmentary and very poor. Aims of this prospective observational study were to evaluate the safety and the efficacy of ultrasound-guided insertional CVC in patients with cancer in palliative phase to confirm the utility of this procedure in this setting of patients, and to evaluate local or systemic infection, CVC related deep venous thrombosis (DVT) and lifespan of the CVC.

## Material and Methods

Adult patients with advanced cancer admitted to the palliative care program of the oncology-hematology department, hospital of Piacenza, North Italy, and requiring an indwelling CVC, were offered the oppurtunity to partecipate in the study. There were 101 women (48.79%) and 106 men (51.21%) with a mean age of 67.68 years old (range 22-86).

No exclusion criteria were contemplated except patients’ refusal. The study was approved by the local ethic committee, as prospective observational study and all the patients gave informed written consent before enrollment. One type of CVC was employed: single lumen 16 gauge Becton Dickinson (Singapore). Each CVC-positioning is considered a single procedure for the study; so a patient who need catheter insertion more than one time, was registered as a new procedure every time CVC is inserted again.

The indications of CVC were: parenteral nutrition and hidratation in a palliative program; patient candidates to chemotherapy, allogenic, autologous transpalantation and procedures other than palliative care only were excluded from this study since they were enrolled in a different study [28].

Operators included two physicians and a nurse, having specific experience with ultrasound and ultrasonically guided procedures, so that the level of experience of the operators would not bias results.

Patients were postured in the Trendelenburg’s position with the head rotated toward the opposite side. The CVC was routinely implanted on the right side; however, if the conditions were unsuitable for implantation on this side, in case of lynphoadenopathy, or postradiation therapy area, or at patient’s request, the CVC was placed on the left side. All procedures were performed using standard aseptic technicques and a local anesthesia with vary small, 22-gauge needle for the venipuncture was applied under ultrasound guidance.

The ultrasound examination were performed using ESAOTE (Genova, Italy) equipped with two transducer between 3.5 to 7.5 MHZ, with a needle guide, whitout a sterile cover. The method that we commonly use is “the three-handed method” [29], this method requires an assistent to hold the probe, while the operator controls the needle and performs the procedure under real-time guidance, and nurse helps the two physicians during the maneuver.

The central vein was identified along its greater longitudinal axis and its relationship with other anatomical structures using Valsalva’s maneuver wich determines an increase of the veins diameter. Under ultrasound-guide in real time, a 16 gauge needle is introduced in the last portion of internal jugular vein. This vein was reached throughout the transduced placed at the point of insertion of sternocleidomastoid muscle into the clavicular, the correct introduction of the needle was always confirmed by ultrasound guidance and by the easy aspiration of venous blood.

Every procedure was scheduled in order to register patient’s data, pathological diagnosis, indications for CVC insertion, type of CVC, number of attempts and early complications if any failure, the procedures have been observed by an independent team. Medications, CVC related blood stream infection, symptomatic deep vein thrombosis and CVC removal or substitution were also recorded. Within two hours after each procedure, chest radiography and ultrasound scanning were carried out to exclude pneumotorax and to evaluate correct catheter position. At the end of treatment or when required, after the removal of the catheter, the tip was sent to the laboratory for bacteriological examination.

No systemic prophylaxis against deep vein thrombosis was adopted and no antibiotic profylaxis was made. Each catheter, at the end of its routinely use, was flushed with 20 mL sterile normal saline, then 5 mL heparinized saline (50 IU/mL). Follow-ups for each patient were scheduled every 10 days, by the home assistance team, until removal of the CVC. The follow up consisted in: patient’s clinical examination, catheters flushing with heparinized solution and catheter exit side dressing.

### Assesment of endpoints

The primary endpoints were: number of pneumotorax, accidental arterial and nerve puncture, major bleeding, number of attempts, failure, local haematoma.

Secondary endpoints were: symptomatic vein thrombosis of upper limbs (early or late), infections, malfunctioning and lifespan of the CVC.

In case of clinical suspicion, of venous thrombosis by progressive arm or facial swelling, ultrasound criteria considered to show the presence of catheter-related thrombosis included visualization of thrombus, absence of spontaneous flow, dilatation of the vein by the Valsalva maneuver and compressibility of the jugular vein as previously reported [30, 31]. Infections were defined as catheter-related bacteremia: isolation of the same organism from catheter, more than 15 colony-forming-units (CFUs) and blood culture, without clinical signs of infections and catheter-related septicemia: isolation of the same organism from catheter more than 15 CFUs and blood culture, with clinical signs of infections [30]. Infections with a clinically apparent focus other than exit site or catheter were excluded. Catheter malfunctioning occlusion was registered when presented.

### Statistical analysis

Demographic data and clinical features were analyzed using descriptive methods. Quantitative variables were summarized using mean and standard deviation. Categorical variables were summarized as counts and percentages. Baseline analysis included all enrolled patients. Statistical tests were performed with Statview Software, last version.

## Results

From 30 October 2000 to 31 October 2008, 209 CVC insertional procedures were applied in 207 patients. The procedure was performed 9 times in hematologic malignancies and 200 times in solid tumors (Table 1); the majority of patients with solid tumors had gastrointestinal cancer and the majority of patients with hematologic malignancies had lynphomas (Table 2). Primary end points: a single needle puncture of the vein was performed on 206 of 209 procedures and one attempt among 209 failed (0.5%) so the procedure revealed to be efficacious in (98.6%) of cases. Failure was caused by vein collapse. There were no differences in mechanical complication, malposition and malfunctioning of CVC with the right (95.7%) and left (4.3%). No bleeding, no nerve puncture and no pneumothorax were reported (Table 3).

**Table 1:** Patients Characteristics

	Number of patients	Percentage of patients (%)
Total	207	100
Median age	67-68 years (range 22-86)	
Gender		
Male	106	51.21
Female	101	48.79
Type of cancer		
Solid tumor	200	96.6
Hematologic malignances	7	3.4

**Table 2:** Types of Cancer

	Number of procedures	Percentage of cancer (%)
Total	209	100
Solid tumor	200	95.7
Gastrointestinal	130	62.2
Lung	10	4.8
Breast	20	9.6
Ginecological cancer	40	19.1
Hematologic malignances	9	4.3
Lymphomas	7	3.3

**Table 3:** Results of Ultrasound Guided Catheter Insertion, Primary End Points and Mechanical Complications

	Number	Percentage (%)
Total procedures	209	100
Access with one attempt	206	98.6
Access with two attempt	2	0.96
Pneumothorax	0	0
Major Bleeding	0	0
Arterial puncture	0	0
Failure	1	0.44
Local Hematoma	0	0
Nerve puncture	0	0

### Secondary end points

Catheter related infection occurred in 2/209 (0.96%) of the catheters inserted; the organisms were Escherichia coli in the two patients and antibiotic therapy resolved the infections.

Symptomatic deep vein thrombosis of upper limbs developed in 1 case (0.46%) and treatment with low weight heparin resolved the thrombosis.

The mean lifespam of CVC was 92.5±9.1 days (range 8-128).

## Discussion

Patients with advanced incurable cancer and malnutrition have been treated nutritionally with intensified oral enteral nutrition (EN), however, when advanced cancer is accompained by obstructions of the intestinal tract and survival depends on nutritional support only total parenteral nutrition and hidratation can became mandatory [32, 33].

We are aware that the goals of care for terminal cancer patients should be refocused on the promotion of quality of life and preparation for death, rather than making every effort to improve the status of nutrition or hydratation as previously reported [34]. However parental hydratation decreased symptoms of dehydratation in terminally ill cancer patients [35]; on the other hand there is a group of patients who, although they are not candidates for any antineoplastic therapy, are still in good physical and mental condition, with expected life spans of three months or more, suffering from conditions such as intestinal obstruction, fistulas or any condition which makes the preferred route of enteral nutrition impossible. The decision of parenteral nutrition should be taken after careful multidisciplinary discussion; patient and caregivers should be aware that PN is not a cancer’s specific treatment and probably will not prolong the patient’s life and it is best if provided at the patient’s home [36]. Patients with advanced and incurable cancer are very frail and every effort may be done to avoid any iatrogenic complications such as complications related to CVC positioning.

Central venous cannulation can be unsafe: the National Confidential Enquiry into perioperative deaths has reported one death resulting from a procedure induced pneumothorax [37].

It must be emphatized that less serious, but costly for patient discomfort, clinician time, hospital stay, economic costs are varying rates for failure and complications from central venous cannulation. Ten of 328 oncologic patients (3.4%) developed pneumothorax after central venous access implanted without US guidance and 6 of them (60%) required tube-thoracostomies [31].

More recently, the etiology and incidence of iatrogenic pneumothorax which can develop after invasive procedures performed for diagnostic and for therapeutic purposes has been reported [38]: the most frequent procedure type causing pneumotorax was central venous catheterization, with 72 patients (43.8%) of the series of 164 patients developing iatrogenic pneumothorax.

In addition, complications are more frequent when more needle passes are necessary because of anatomical variation or difficult veins (small veins, not palpable landmarkers). Anatomical variations of the internal jugular vein were found in 8% of patients studied with ultrasound-guided CVC [39]; the easiness and the shorter time required to perform ultrasound-guided catheterization, together with the higher rate of success and decreased incidence of complications, make using the ultrasound-guided CVC preferable to conventional CVC [40-42].

We recently reported a very large series of 1978 CVC insertional performed procedures with ultrasound guidance in 1660 consecutive patients suffering from cancer undergoing chemotherapy or bone marrow transplantation, none of the patients experienced major complications (pneumotorax, hemothorax, nerve lesions) [28].

In the present study ultrasound-guided CVC procedures were performed by our own specifically experienced oncologists and hematologists, and we agree with recent report [29] that ultrasound is an easily learned technique that not only enhances the physical examination, but has the distinct advantages of being a portable tool that can provide real-time guidance for CVC insertion and other interventional procedures such as biopsy, abscess drainage, paracentesis, thoracentesis etc and for critically ill and patients with advance disease the procedure can be performed easily at bedside or at home as previously reported [22-28]. Physicians with specific experience for ultrasound guided CVC insertion procedure were defined operators who have performed > 50 CVC insertion under ultrasound guidance [5, 29].

It must be emphasized that the use of ultrasound is not limited to radiologist; the American Medical Association policy privileges the ultrasound imaging technique diffusion in medical practice [43], the American College of Emergency Physicians and the American College of Surgeons support the use of ultrasound by members of their societies and address ways to obtain and maintain competency as well as ensuring quality control [44, 45].

We believe that this technique is very useful not only in the oncology and haematology department, for CVC placement, staging, disease control, follow-up, ultrasound guided-procedures [22-28], but also in the palliative setting as recently reported.

### Conclusion

Our study demonstrated ultrasound guided CVC insertion is a safe and effective technique, also in a setting of frail patients with advanced and incurable cancer: it allowed home parental nutrition and hydratation in 206/207 patients (98.6%). We believe this technique can be applied easily to most practitioners in clinical oncology for patients in palliative program with relevant patient’s benefits.
